# Seasonal Variation of Rhegmatogenous Retinal Detachment: A Systematic Literature Review

**DOI:** 10.7759/cureus.92028

**Published:** 2025-09-11

**Authors:** Christos Tooulias, Dimitrios Papaconstantinou, Konstantinos Droutsas, Angeliki Androu, Paraskevi Theofilou, Panagiotis Theodossiadis, Panagiotis Stavrakas, Ilias Georgalas

**Affiliations:** 1 2nd Department of Ophthalmology, University of Athens Medical School, "Attikon" University General Hospital, Athens, GRC; 2 1st Department of Ophthalmology, University of Athens Medical School, General Hospital of Athens "G. Gennimatas", Athens, GRC; 3 1st Department of Ophthalmology, General Hospital of Athens "G. Gennimatas", Athens, GRC

**Keywords:** air temperature, atmospheric pressure, humidity, meteorological factors, rhegmatogenous retinal detachment (rrd)

## Abstract

Rhegmatogenous retinal detachment (RRD) is a serious ocular emergency often linked to retinal tears and vitreoretinal traction. While established risk factors include age, myopia, and posterior vitreous detachment (PVD), the role of environmental variables remains unclear. The aim of this literature review is to investigate the seasonal variation of RRD across different climates and geographical regions. By reviewing and synthesizing the latest findings, this study seeks to enhance understanding of the seasonal patterns of RRD and to inform the development of preventive strategies aimed at addressing or reducing its incidence. A systematic literature review was conducted using MEDLINE, Scopus, and Cochrane Library databases. Keywords related to RRD and climatic factors were used. Inclusion criteria focused on observational studies published in English within the past decade, involving adult populations. Studies were screened by title, abstract, and full text, with reference lists reviewed for additional sources. All studies employed a retrospective review methodology and were conducted across two continents: Asia (62.5%) and Europe (37.5%). Two studies encompassed entire countries. The patient populations typically had a mean age ranging from 50 to 69 years, with a higher incidence of RRD observed in males. Most studies quantified RRD cases within specific time intervals, while two studies calculated incidence rates relative to the sample size. Four studies reported a statistically significant seasonal association, with the highest incidence observed in the summer months. One study identified a significant correlation in March, while another reported a peak month in April and October. Two studies found no significant seasonal correlation. Regarding meteorological factors, the findings were inconsistent: increased solar radiation was associated with higher rheumatogenous detachment incidence, while daylight hours exhibited a bimodal distribution. Low atmospheric pressure was correlated with higher occurrence, although no single factor emerged as consistently predictive. This review suggests a possible seasonal pattern in RRD incidence, with summer being the most commonly associated season, though findings were not consistently statistically significant. PVD, a known precursor to retinal detachment, was identified as a major risk factor; however, only one study examined its seasonality, finding no significant association. Other well-established risk factors, such as myopia, age, and male sex, were also confirmed. Associations with meteorological factors - especially low atmospheric pressure, temperature, and humidity - were observed in some studies but remain inconclusive. The retrospective design and lack of control for seasonal population shifts or trauma-related detachments limit generalizability. This review suggests a possible seasonal pattern in RRD incidence, particularly during summer, though findings remain inconclusive. Recognized risk factors such as myopia, age, and PVD are reinforced. Further research is needed to clarify environmental influences using more robust, prospective study designs.

## Introduction and background

Rhegmatogenous retinal detachment (RRD) refers to a pathological condition in which the neuroretina detaches from its underlying layer - the retinal pigment epithelium (RPE) - due to the presence of a retinal tear in combination with vitreoretinal traction. This disruption allows the accumulation of liquefied vitreous beneath the neuroretina, leading to its detachment from the underlying choroid. RRD is an acute and serious retinal disease, the treatment of which usually dictates a surgical approach [1]. Predisposing factors include advanced age, male gender, proliferative diabetic retinopathy [1], myopia [2], previous complicated cataract surgery [2], ocular trauma [3], posterior vitreous detachment (PVD), and its structural changes [1]. However, environmental triggers leading to the phenomena cascade culminating in RRD remain largely under investigation, with the exception of temperature, atmospheric pressure, and solar radiation [4-6]. Identifying such factors is crucial for understanding the pathophysiology of RRD, but also for risk awareness and the establishment of prevention strategies.

The influence of seasonal variation or meteorological factors on the prevalence of RRD remains unclear. To date, the role of seasonal variation in RRD has been reported in several studies [7-12]. In particular, the prevalence of RRD is reported to peak during the summer months but reaches a minimum during winter [7-10,13], speculating that specific climatic factors might significantly influence its seasonal distribution [7]. For instance, increasing light intensity has been reported to correlate with higher RRD incidence [7], while other authors advocate for an association between higher average temperature and increased RRD occurrence [14]. However, in other studies, no seasonal variation was found [11-17] or even noted a winter peak and a summer nadir [18]. Although climatic parameters may be important for the development of RRD, it is not yet clear whether there is a seasonal association with its occurrence, since light intensity and temperature are climatic factors that are associated with increased levels of outdoor activity, which could be at least partly responsible for its increased occurrence.

The purpose of this literature review is to investigate the possible association of climatic factors, such as mean monthly temperature, relative humidity, and hours of sunshine, with monthly RRD incidence worldwide.

## Review

Methods

This literature review was conducted in accordance with the guidelines of Pare et al. [18]. The MEDLINE (Medical Literature Analysis and Retrieval System Online), Scopus, and Cochrane Library bibliographic databases were used. The following keywords were used to identify relevant scientific publications: “rhegmatogenous”, “retinal detachment”, “climatic influence”, “seasonal variation”, and “meteorological factors”. Keywords were searched as part of the text in the main title, in the abstract, or within the text. At the same time, the terms “and” and “or” were used as auxiliaries in search engines. Keywords were used separately, but also in combination, while the literature search was based on observational studies published within the last decade, published in English, and conducted in adult populations (≥18 years), regardless of gender.

The initial screening was performed based on titles. Articles deemed irrelevant to the review objective were excluded. Abstracts of the remaining studies were subsequently assessed, and those not meeting the inclusion criteria were also excluded. Eligible full-text articles were then evaluated for relevance and completeness of data. Studies lacking sufficient information regarding the topic or aim of the review were excluded. Finally, reference lists of the included articles were manually screened to identify additional eligible studies.

Results

**Figure 1:**
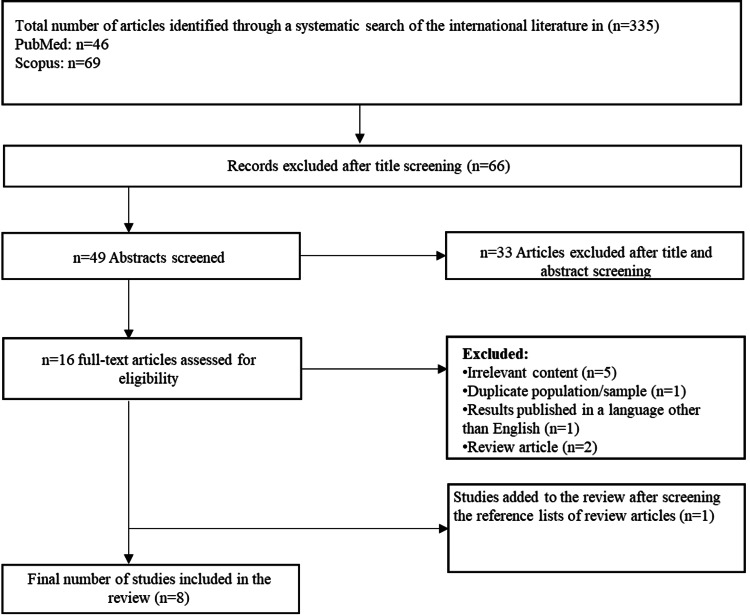
Flow diagram of the selection procedure.

Title search, as described, led to the identification of 335 studies. From these, 286 studies were rejected after abstract investigation, and 28 studies were rejected after text scrutiny. Finally, only eight studies met the selection criteria. One additional study was included following reference list screening, according to the same criteria. Figure 1 reports the main characteristics of the studies included in the present review.

All selected studies employed a retrospective review for data collection and analysis. Geographically, the studies were distributed across two continents: Asia (62.5%) and Europe (37.5%).

Demographic analysis of the patient populations in these studies revealed a consistent age distribution, with most reporting a mean age between 50 and 69 years, which aligns with the known pathophysiology of RRD. Regarding sex distribution, all studies indicated a higher incidence of RRD in males. The predominant approach to estimating RRD incidence involved counting the number of cases within defined time intervals (e.g., months, quarters, seasons). In contrast, a minority of studies calculated incidence rates as a proportion of cases relative to the population size.

Among the eight selected studies, four reported a statistically significant association between the occurrence of RRD and season. Most studies observed the highest incidence of RRD during the summer months, with some indicating an overlapping period from spring through autumn. Table 1 summarizes the findings of these studies. Two studies reported no significant relationship between seasonal or weather-related variables and RRD incidence [19,20]. Kim et al. identified April and October as peak months for RRD incidence [21]. Only one study, by Choo et al. [22], reported a significant seasonal correlation in spring, particularly in March. Notably, Prabhu et al., in a unique approach, defined the summer period as March through May and found a significant association within this timeframe [10]. Notably, only two studies extended their scope to cover an entire country [23,24].

**Table 1:** Summary of the studies' results. RRD: Rhegmatogenous retinal detachment

Study (Author/year)	Country	Patients (Eyes)	Study period	Peak season/month of RRD	Lowest season/month of RRD	P value
Prabhu et al. [10], 2016	India	76 (76)	2012-2014	Spring (March, April, May)	Monsoon season (June, July, August)	>0.05
Sevillano Torrado et al. [6], 2019	Spain	256 (256)	2008-2014	June-July	November	n/a
Erdöl et al. [19], 2020	Turkey	276 (281)	2011-2018	September	December	>0.05
Kim et al. [21], 2020	Korea	1394 (974)	1996-2016	April and October	August	n/a
Iida et al. [21], 2021	Japan	543 (571)	2015-2019	July	February	>0.05
Choo et al. [22], 2021	Korea	32,088 (39,410)	2014-2017	March	February	<0.001
Ben Ghezala et al. [23], 2022	France	101,085	2010-2016	June-July	August, February	<0.001
Xu et al. [24], 2023	China	3629 (3629)	2015-2019	April	November	n/a

Some of the reviewed studies also examined secondary objectives, such as the potential influence of various meteorological factors (e.g., ambient temperature, atmospheric pressure, daylight duration, humidity, and precipitation) on RRD incidence. However, the findings were inconsistent. For instance, Sevillano Torrado et al. observed that increased solar radiation was associated with higher RRD incidence, suggesting that solar radiation may contribute to PVD, and consequently, to RRD [6]. In contrast, Choo et al. identified a bimodal distribution of RRD incidence in relation to daylight hours, with peaks at specific times of the year [22]. Additionally, Iida et al. reported an inverse relationship between atmospheric pressure and RRD incidence, noting higher occurrence under low-pressure conditions [20]. Nonetheless, no single meteorological factor has consistently emerged as a significant predictive variable across studies. This highlights the need for further research to clarify these complex associations and to evaluate the potential inclusion of environmental factors in RRD risk assessment and prevention strategies.

Discussion

The aim of this literature review was to examine the relationship between RRD and climatic or other seasonal factors through a comprehensive analysis of relevant studies. Understanding this relationship could contribute to the development of new and improved strategies for managing patients with RRD by incorporating related risk factors.

This review identified eight studies conducted over a 10-year period (2014-2024), spanning two continents (Europe and Asia). According to the literature, summer appears to be the season most frequently associated with RRD. However, due to the limited number of studies and the proximity of statistically significant results to non-significant findings, further research is warranted to clarify this association.

An additional key finding involves the seasonality of PVD, which often precedes RRD [1]. All studies in this review acknowledged PVD as a risk factor for RRD, though only the study by Prabhu et al., conducted in India, analyzed PVD as a distinct variable [10]. Based on data from 76 RRD cases over two years, their study found a non-significant association between season and PVD occurrence. More specifically, the highest RRD incidence was observed during the warmest months (temperature range: 25-34 °C), which, however, did not reach statistical significance when compared to its incidence during the coldest months (temperature range: 23-30 °C) [10]. India’s climate is tropical with an average annual temperature of 24-28 °C, while three seasons are mainly distinguished: the mild and rainless winter, the hot and dry spring, and the humid, tropical summer. In the same study, an attempt was made to determine whether the shrinkage of the vitreous due to excessive sweating and dehydration in hot climates can contribute to the occurrence of retinal detachment during summer months, as changes in the intravascular and extravascular compartments may have an impact on the intraocular pressure and vitreous volume. More specifically, a comparison of the mean intraocular pressure of the affected eye with that of the other eye yielded a non-statistically significant difference in values. However, the authors advocate that the increased incidence of RRD during warm, humid seasons and the association with low rainfall and relative humidity underscore the importance of seasonality. Future research may benefit from treating PVD as an independent variable to better assess the seasonal pattern of RRD, given the strong temporal and pathophysiological link between the two conditions. Future research may benefit from treating PVD as an independent variable to better assess the seasonal pattern of RRD, given the strong temporal and pathophysiological link between the two conditions.

Beyond seasonal correlations, several included studies highlighted established risk factors for RRD, such as myopia and older age. These associations are well documented, as the pathophysiology of RRD connects these factors to disease development. Specifically, myopic eyes exhibit increased posterior segment volume, which can lead to greater vitreoretinal traction [25]. A longer axial length further elevates the risk for PVD and subsequent RRD. Additionally, age-related vitreous degeneration likely contributes to increased RRD risk. Male sex also appears associated with higher RRD incidence, possibly due to generally longer axial eye length in men [25].

Findings related to meteorological factors and RRD incidence varied. Lower atmospheric pressure was negatively associated with RRD occurrence [20]. Specifically, in this study by Iida et al. in Japan, the seasonal RRD incidence was the highest in summer and the lowest in winter, with no statistically significant difference between them. However, an important result of this study was the statistically significant negative correlation between the average daily variation of seven meteorological parameters (mean temperature, maximum temperature, maximum wind speed, maximum instantaneous wind speed, humidity, mean barometric pressure at sea level and minimum barometric pressure at sea level) with the incidence of RRD. In an earlier study, also conducted in Japan, RRD occurrence was found to be higher in early summer (May-June) and less frequent in winter (January-February), while its frequency was significantly correlated with increased sunshine hours and average temperature. Temperature, humidity, hours of sunlight, and precipitation also showed statistically significant associations with RRD incidence in some studies, highlighting the need for further investigation to confirm these relationships [17].

Most studies reviewed were retrospective in design - a common approach due to its relative cost-effectiveness and practicality, though not without limitations. A key limitation was the lack of adjustment for population fluctuations and seasonal changes in patient volumes. Patient movement between regions or healthcare centers may affect data consistency. Additionally, many studies did not distinguish between RRD and non-RRD. This distinction is critical, as ocular trauma - a known risk factor for both traumatic and rhegmatogenous detachment - may be more frequent during warmer months due to increased participation in recreational activities [26-30]. This could confound the seasonal variation observed in RRD incidence without a clear link to meteorological conditions. Finally, the number of included studies is also limited due to conformity to our study’s strict inclusion criteria, constituting, however, an additional limitation.

## Conclusions

This literature review highlights a potential seasonal pattern in the incidence of RRD, with summer emerging as the most commonly associated season. However, current evidence remains inconclusive due to methodological limitations and inconsistent findings across studies. The review reinforces established risk factors, such as myopia, older age, and male sex, and underscores the importance of posterior vitreous detachment as a precursor to RRD. Although some studies identified associations between meteorological variables and RRD incidence, further research is required to clarify these links. Future investigations should adopt prospective designs, account for population fluctuations, and differentiate between types of retinal detachment to better understand the role of environmental and seasonal influences in RRD development.
